# Mesophilic microorganisms build terrestrial mats analogous to Precambrian microbial jungles

**DOI:** 10.1038/s41467-019-11541-x

**Published:** 2019-09-20

**Authors:** N. Finke, R. L. Simister, A. H. O’Neil, S. Nomosatryo, C. Henny, L. C. MacLean, D. E. Canfield, K. Konhauser, S. V. Lalonde, D. A. Fowle, S. A. Crowe

**Affiliations:** 1 Departments of Microbiology and Immunology and Earth, Ocean, and Atmospheric Sciences, University of British Columbia, Vancouver, Canada; 2 Nordic center for earth evolution (NordCEE), University of Southern Denmark, Odense, Denmark; 3 Healthy Waterways, Brisbane, Australia; 4 Research center for Limnology, Indonesian Institute of Sciences (LIPI), Jawa Barat, Indonesia; 5 GFZ German Research Centre for Geosciences, Potsdam, Germany; 6 Independent researcher, Halifax, Canada; 7 Department of Earth and Atmospheric Sciences, University of Alberta, Edmonton, Canada; 8 European Institute for Marine Studies, Technopôle Brest-Iroise, Plouzané, France; 9 Department of Geology, University of Kansas, Lawrence, KS USA

**Keywords:** Biogeochemistry, Climate sciences

## Abstract

Development of Archean paleosols and patterns of Precambrian rock weathering suggest colonization of continents by subaerial microbial mats long before evolution of land plants in the Phanerozoic Eon. Modern analogues for such mats, however, have not been reported, and possible biogeochemical roles of these mats in the past remain largely conceptual. We show that photosynthetic, subaerial microbial mats from Indonesia grow on mafic bedrocks at ambient temperatures and form distinct layers with features similar to Precambrian mats and paleosols. Such subaerial mats could have supported a substantial aerobic biosphere, including nitrification and methanotrophy, and promoted methane emissions and oxidative weathering under ostensibly anoxic Precambrian atmospheres. High C-turnover rates and cell abundances would have made these mats prime locations for early microbial diversification. Growth of landmass in the late Archean to early Proterozoic Eons could have reorganized biogeochemical cycles between land and sea impacting atmospheric chemistry and climate.

## Abstract (web summary)

Microbes venturing onto land could have impacted biogeochemical cycles billions of years before terrestrial plants, but insight into this process on ancient Earth has remained elusive. With the discovery and analysis of microbial mats analogous to those of the Precambrian, Finke and colleagues infer how these microbial jungles likely shaped ecology and climate.

## Introduction

Modern global biogeochemical cycles are distributed across the terrestrial and marine realms. Half of the global primary production, for example, is from algal and cyanobacterial growth in the sunlit surface ocean, while the other half comes from plants inhabiting the continents^1^. More generally, global nutrient cycles are characterized by extensive links between terrestrial and marine ecosystems, forming multiple feedbacks that regulate biological production and climate over geological time-scales^2^. Such biogeochemical feedbacks between land and ocean have operated at least since the emergence of terrestrial plants some 400 million years ago^2,3^, but may have begun much earlier during the Precambrian Eons, when microbial mats developed on land hundreds of millions or even billions of years before the evolution of plants^4–8^. Aquatic biofilms, hypersaline, and marine microbial mats are relatively abundant today, e.g. ^9–18^, and these provide insight into the ecology and biogeochemistry of prokaryote-dominated ecosystems. Subaerial examples, however, with the capacity to broadly colonize the early continents^8^, have remained elusive on the modern Earth. Indeed, large-scale colonization of land by microbial mats today is precluded by both animal predation and strong competition with plants for photosynthetic niche-space^19,20^. The lack of information on the ecology, physiology, and biogeochemical functioning of terrestrial microbial mats leaves models for the possible role of such ecosystems in the Precambrian Earth system almost entirely conceptual. Such information, in principle, could be obtained through the study of modern terrestrial mats with features analogous to those inferred from the remains of terrestrial ecosystems preserved in the rock record.

**Fig. 1:**
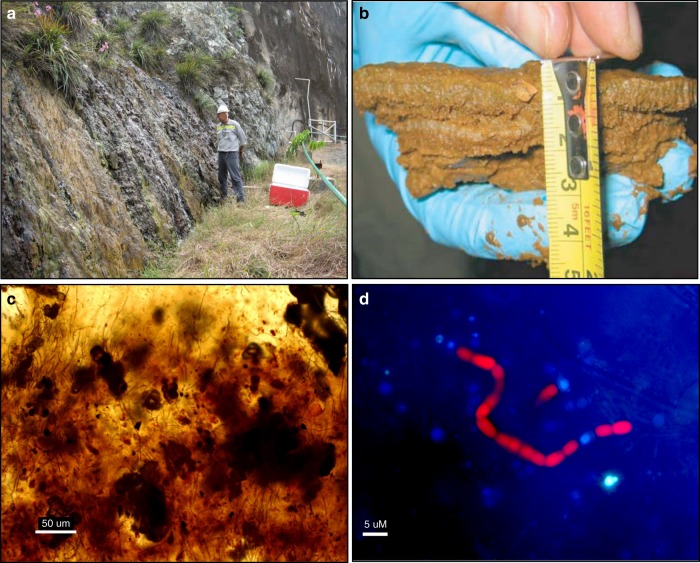
Images of the Balambano mat. **a** Field site with microbial mat growing on the outcrop of ultramaphic rock. **b** Cross section of a freshly sampled microbial mat showing the horizontal layering of the mat and a green, photosynthetic layer close to the mat surface. The orange colour is likely due to the presence of iron(oxy)hydroxides. **c** Thin section of the microbial mat showing trapped opaque minerals suspended in a translucent matrix containing abundant microbial filaments. **d** Fluorescence microscopy image of filamentous cyanobacteria in the mat showing presence of heterocystous cyanobacteria, red is from chlorophyll autofluorescence and the heterocyst is identified by its lack of chlorophyll

We discovered a subaerial microbial mat growing on exposed ultramafic rock surfaces at the Balambano Hydroelectric Dam on Sulawesi Island, Indonesia (Supplementary Fig. 1). The mat grows on steep outcrops tens of square metres in size (Fig. 1a), accretes to thicknesses up to 6 cm, and has many chemical and biological features that are comparable to the terrestrial microbial communities found in Precambrian rocks^4–8^. Geochemical and microbiological analyses reveal that the mat hosts a diverse photosynthetic microbial community with the metabolic capacity to drive large-scale biogeochemical cycling of C, N, and other elements released during rock weathering. Quantitative, model-based reconstructions of the possible role of similar such mats during the Precambrian Eons imply that terrestrial ecosystems could have driven fluxes of matter and energy at global scales, promoted biological evolution, and helped regulate climate well before the emergence of land plants in the Phanerozoic Eon.

## Results

### Microbial community composition

**Fig. 2:**
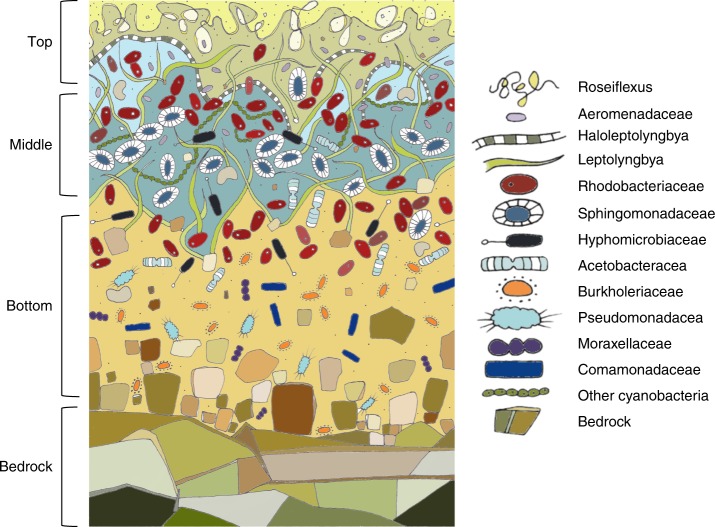
Stylized illustration of Balambano microbial mat. The illustration depicts the positions of major microbial taxa, weathered rock fragments, and underlying bedrock. Cells not drawn to scale

**Fig. 3:**
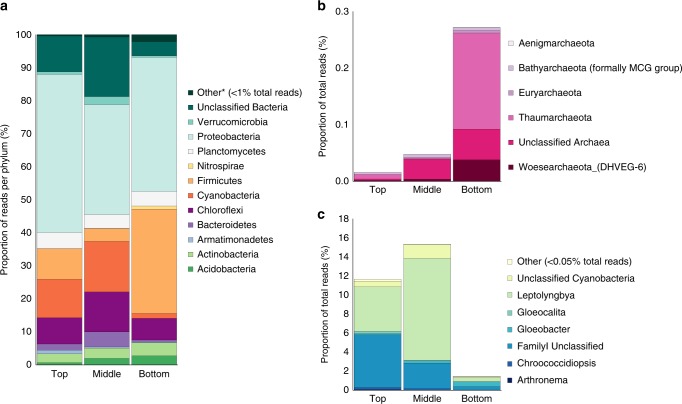
Composition of the Balambano mat microbial community. Distribution of Illumina 16S rRNA gene sequences are shown per bacterial phylum **(a**), per archaeal phylum **(b**) and per cyanobacterial genera (**c**), in three sections of the microbial mat (top = 0–2 mm; middle = 2–5 mm; ottom = 5–20 mm). The number of sequence reads per phylum is calculated as a percentage of the total sequence reads for each sample. *Other represents reads that contributed <1 and 0.05% of the total number of reads for A and C, respectively

The Balambano microbial mat supports a taxonomically diverse assemblage of microorganisms. It is compositionally layered (Figs. 1b and 2), and is largely comprised of filamentous bacteria and their exopolymers, as well as suspended mineral grains (Figs. 1c, d and 2). Cell abundances range from 10^8^ to 10^9^ cells cm^−3^ and decrease with increasing depth in the mat (Supplementary Table 1). The mat is made up of a diverse assemblage of bacteria including an abundance of oxygenic and anoxygenic phototrophs (Fig. 3). Estimates of species richness are on the order of 3000–6000 taxa based on 97% identity between 16S rRNA genes (Supplementary Fig. 2 and Supplementary Table 2)—comparable to phototrophic communities in the sunlit surface ocean^21^ and hypersaline microbial mats^16^, but less than typical soil microbial communities, which can exceed tens of thousands of species^22,23^.

**Fig. 4:**
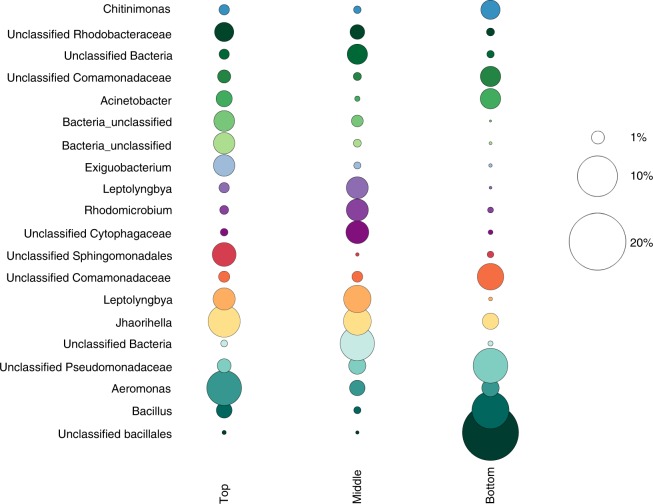
Detailed composition of Balambano microbial mat community. The top 20 most abundant taxa (at 97% sequence identity, commonly accepted as defining the species level), in the top, middle, and bottom microbial mat sections

Beyond information on microbial community diversity, we also identify a number of taxa that can be linked to specific ecological roles. These taxa are reported at the genus level where possible, based on sequence resolution and database matching, and at the family level otherwise. In the upper reaches of the mat, the phototrophic community is dominated by non-heterocystous, filamentous Cyanobacteria (*Leptolyngbya* and *Haloleptolyngbya*) and filamentous, photosynthetic Chloroflexi (*Roseiflexus*, *Chloronema*, and *Candidatus Chloroploca*) (Figs. 1d, 3 and Supplementary Table 3). Heterocystous filaments can also be observed (Fig. 1d) in the upper mat and known heterocystous cyanobacteria including *Anabaena* and *Nostoc* genera (Fig. 3c), which belong to family I cyanobacterial groups, comprise up to 5.5% of the photosynthetic community based on 16S rRNA gene sequences (Fig. 3; Supplementary Table 3). Cyanobacteria in the mat derive from some of the deepest branching cyanobacterial lineages including *Gloeobacter*, *Thermosynechococcus* and the filamentous *Pseudanabaena* and *Leptolyngbya* that likely radiated in the Meso- to Neoarchean Eras^24,25^. The base of the mat is enriched in firmicutes, including organisms related to anaerobic *Clostridia* genera and facultative aerobic *Bacilli* genera. Throughout the mat, Proteobacteria dominate (Figs. 3a and 4 and Supplementary Fig. 3), with each layer of the mat hosting taxonomically distinct populations. At the family level, *Rhodobacteraceae*, *Aeromonadaceae*, and *Sphingomonadaceae* are most abundant in the upper mat, *Rhodobacteraceae*, *Acetobacteraceae,* and *Hyphomicrobiaceae* dominate in the middle mat, and *Pseudomonadaceae*, *Comamonadaceae*, *Moraxellaceae,* and *Burkholderiaceae* are most abundant in the bottom layer (Figs. 3a and 4, Supplementary Fig. 3, and Supplementary Table 3). Lineages of the Alpha- and Gammaproteobacteria with metabolic potential for N_2_ fixation (genera *Rhizobium*, *Azospirillum*, and *Azomonas*) are also present (Supplementary Table 3). Nitrification, furthermore, is implied by the presence of bacteria from the family *Nitrosomonadaceae* (genus *Nitrospira* (Supplementary Table 3)), which are known from lab cultures as obligate nitrifiers^26^. Also identified were members of the phyla Verrucomicrobia (genus *Candidatus* Methylacidiphilum) and Alphaproteobacteria (family *Methylobacteriacea*) (Supplementary Table 3), both known to grow through methylotrophy or methanotrophy^27^. The archaeal phyla Thaumaracheaota, Bathyarchaeota, and Euryarchaeota were also present (Fig. 3b and Supplementary Table 3), suggesting metabolic potential for archaeal nitrification (genus *Candidatus* Nitrososphaera)^28,29^ and methanogenesis or C1 compound metabolism (genera *Methanomethylovorans* and phylum Bathyarchaeota)^30,31^, respectively. These subaerial mats thus support taxonomically and metabolically diverse communities with the capacity for biogeochemical cycling of carbon and nitrogen distributed across multiple microbial taxa.

### Chemical composition

The Balambano mat microbial community retains bedrock components in the form of mineral grains and amorphous precipitates (Figs. 1c and 2, and Supplementary Fig. 4), which are trapped in the organic matrix of filamentous cells and extracellular polymeric substances (EPS). Such multicellular filaments of cyanobacteria and chloroflexi often stabilize microbial mats. When viewed in cross-section (Fig. 1b, c), the mat exhibits millimeter-scale laminations as well as finer-scale structural features, which include vertically aligned filaments and clusters of opaque inorganic material (Fig. 1c), presumably iron (oxyhydr)oxides. The inorganic chemical composition of the mat (Supplementary Table 4) is mostly Fe, Si, and Mg, with a mineralogy dominated by X-ray amorphous phases (Supplementary Fig. 4), but also sheet silicates such as nontronite {Ca_O.1_Fe_2_(Si,Al)4O_10_(OH)_2_-4H_2_O} and pimelite {Ni_3_Si_4_O_10_(OH)_2_-5H_2_O}. The presence of clay minerals in the mat confirms its capacity to trap and retain material to form protosoil. Organic carbon concentrations are highest (12 wt.%) in the upper 2 mm of the mat, where the isotopic composition of organic C (δ^13^C) is −33‰ (Supplementary Fig. 5), indicative of photosynthetic C fixation^32^. Deeper in the mat, organic carbon concentrations decline to 1 wt.%, with isotopic compositions as light as −41‰ (Supplementary Fig. 5), indicating incorporation of ^13^C-depleted carbon, likely through methanotrophy. With 10^8^–10^9^ cells cm^−3^ cellular biomass makes up from 0.001 to 20% of the total organic C in the mat (see Methods below). Organic N reaches nearly 1 wt.% in the surface of the mat with a C:N ratio of 15—higher than the Redfield ratio of around 6 that typifies marine phytoplankton today^33^ and in the past (Supplementary Fig. 5)^34^. The N isotopic composition of organic matter (OM) ranges from −3.4 to −0.6‰ (Supplementary Fig. 5), indicative of biological fixation of atmospheric N_2_ (ref. ^35^).

### Biological production and growth rate

**Fig. 5:**
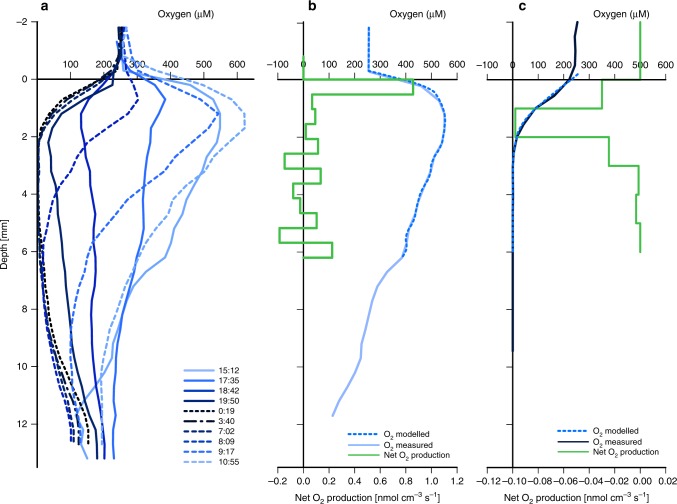
Profiles of oxygen concentrations as a function of depth in the mat. **a** Diel oxygen profiles measured in the greenhouse every 2 h. **b** Net oxygen production modelled from the 15:12 profile from **a** using a 1D transport-reaction model^72^. **c** Net oxygen production modelled from the 3:40 profile from Fig. 3a using a 1D transport-reaction model^72^. Respiration is restricted to the oxic surface layers. High oxygen concentrations during the day likely stimulate respiration throughout the mat. The areal rates calculated from the rate profiles are presented in the box model (Supplementary Fig. 5) and used for the calculations in Table 1 and Fig. 6

Physiological measurements and mass balance considerations constrain the metabolic potential and overall biogeochemical activity of the Balambano mat. Measurements of photosynthesis revealed net O_2_ production of 23 mmol m^−2^ d^−1^ (Fig. 5 and Table 1), while mass balance calculations of mat growth suggest in situ organic carbon accretion rates of 20 mmol m^−2^ d^−1^ (Supplementary Fig. 6 and Table 1). Measured CH_4_ production rates were up to 4.1 mmol m^−2^ d^−1^ (Supplementary Fig. 6 and Table 1). Together, these rates indicate that both retention of OM and CH_4_ loss are important sinks of reduced carbon in the mat that balance net O_2_ production (Supplementary Fig. 6 and Table 1).

**Table 1:** Gross and net oxygen production

Single sources and sinks	Net O_2_ production (mmol m^−2^ d^−2^)	Method
1D model	23	1
O_2_ flux atmosphere + rock	8.4	2,3
CH_4_ flux atmosphere + OM production	24	4,5
**Single sources and sinks**	**Gross O**_**2**_ **production (mmol** **m**^**−2**^ **d**^**−2**^**)**	**Method**
1D model net O_2_ production + respiration	38	1,6
O_2_ flux atmosphere + rock + respiration	23	2,3,6
CH_4_ flux to atmosphere + OM production + respiration	39	4,5,6

1, 1D transport reaction based on daytime greenhouse profile; 2, flux determination from daytime greenhouse profile; 3, flux determination of in situ profile; 4, in situ mat accretion; 5, headspace analysis in dark mat incubation; 6, 1D transport reaction modelling based on night time greenhouse profiles, see also Supplementary Fig. 4

*OM* organic matter

## Discussion

Many of the Balambano mat features are similar to the geochemical, mineralogical, fossil, and structural evidence for the presence of microbial life on the continents and early paleosols during the Archean Eon^4,8,36^. The filamentous morphology of the Balambano mat may be essential for the stabilization of subaerial mats and is amenable to preservation as readily identifiable microfossils, such as those found in Precambrian terrestrial rocks^4,8^. In fact, a number of microbial lineages that have been interpreted from Archean paleosols, such as Actinobacteria, purple sulphur bacteria, and methanogenic Archaea^37^, are found in the Balambano mat (Fig. 3). At a larger scale, mineral trapping (Fig. 1c), and growth through the accretion of compositionally distinct layers (Figs. 1b and 2), can leave characteristic laminated features in the rock record akin to structures recognized as stromatolites in Archean terrestrial^38^ and marine^39,40^ rocks. Taken together, the structural support provided by filaments to the Balambano mat and the putative filamentous nature of early terrestrial stromatolite communities suggest that the evolution of multicellularity in the form of filamentous bacteria may have been requisite for the proliferation of thick, subaerial microbial mats and their preservation in the rock record. Photosynthetic and methanogenic organisms in the mat impart characteristic geochemical signals such as ^13^C-depleted OM, as well as elemental stoichiometries, that can ultimately be recorded as fossil OM in paleosols^7^ and fossilized microbial mats^8^. Notably, mat retention of inorganic material (Fig. 1b, c) provides a weathering environment conducive to clay mineral formation and soil accretion^41^—processes critical to early soil development, mineral evolution^42^, and the preservation of weathering products as paleosols. By extension, microbial mats would also have influenced the delivery of weathering products to oceans, and the burial of OM in marine sediments. For example, terrestrial microbial activity has been invoked to explain increased nutrient, sulphate, and trace element fluxes to the oceans in the Archean Eon^5,43,44^, and enhanced delivery of clay minerals to oceans in the Neoproterozoic Era^41^.

Physiological information from the Balambano mats can be used to infer the possible biogeochemical role of similar mats during the Precambrian Eons. We note that modern microbial communities are imperfect analogues to Precambrian microbial communities, as many of the specific taxa that comprise these communities likely arose relatively recently^45^. Nevertheless, most metabolic potential hosted within these taxa emerged early in Earth’s history^46^ and is likely retained at the community level such that the capacity to drive biogeochemical cycling is conserved through time despite taxonomic reshuffling of metabolic potential through horizontal gene transfer^47^. We thus argue that the metabolic potential and biogeochemical functioning of Balambano mat makes a good analogue for similar mats in the Precambrian Eons, but recognize that the taxonomic composition of the modern mat is unlikely to reflect the taxonomic composition of Precambrian mats. Relationships between taxonomy and function remain controversial^48^, but when better known, may prove important considerations for establishing and interpreting analogue systems.

**Fig. 6:**
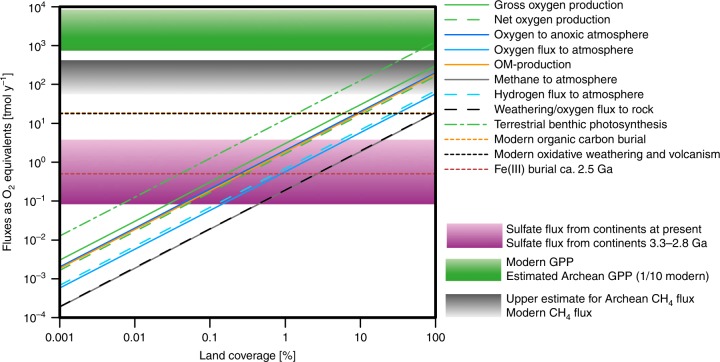
Global elemental budgets and fluxes. Land coverage is based on a total land surface area of 2.5 × 10^7^ km^2^ (ref. ^50^). At about 0.02% land coverage, the measured gross oxygen production could account for the present sulphate flux and would exceed historic values. At a coverage of about 1%, it would match modern oxidative weathering and volcanism, or modern organic carbon burial. A coverage of more than 100% is required to reach the estimated Archean gross marine primary productivity. Shaded areas for sulphate flux, methane flux, and gross primary productivity refer to fluxes ranging from Archean (darker colours) to present (lighter colours) values. Horizontal lines represent global production, diagonal lines represent terrestrial production dependent on land coverage (modified after^44^). Values according to ref. ^44^ were recalculated for 2% of land surface to be covered by rivers and lakes^52^

Rates of photosynthetic productivity and OM accretion in Balambano mat are similar, on an area-specific basis, to moderately productive coastal marine waters or perennial grasslands today^1^, implying that if subaerial mats were widespread on the Precambrian continents, then they would have played a key role in global carbon and oxygen budgets (Fig. 6). Upper estimates for gross marine productivity in the Precambrian Eons are between 170 and 500 Tmol yr^−1^ (ref. ^49^). Respiration in Balambano mat consumes 66% of the O_2_ produced giving a gross production of 38 mmol m^−2^ d^−1^ (Table 1) or 14 Mmol C km^−2^ yr^−1^, similar to values estimated for Archean benthic freshwater mats^44^ and at the lower end of rates (range 6–1200 mmol m^−2^ d^−1^, average 240 mmol m^−2^ d^−1^, Supplementary Table 5) measured in hypersaline, marine, and aquatic environments (Supplementary Table 5). Applying our measured rates of gross productivity to an area equivalent to, for example, 5% of the late Archean land area (2.5 × 10^7^ km^2^ (ref. ^50^), lower end of values reported^51^ for the Neoarchean) yields a terrestrial productivity of 17 Tmol yr^−1^ (Fig. 6), or up to 10% of total estimated global production with minimal coverage of land surfaces. Benthic lacustrine mats could contribute to some of this production, but modern lakes and rivers cover only ~2% of the land surface^52^ and just a small fraction of that is suitable habitat for mat growth (Fig. 6). The fraction of the available land mass colonized by mats would depend on precipitation patterns and water availability. Modern climate and topography allow development of forests and grasslands on ~55% of today’s land surface^53,54^, which when translated to the Archean would have allowed terrestrial mats to contribute up to ~190 Tmol yr^−1^ to global productivity. Landmasses in the tropics today are typically overgrown with dense forest and ‘tangled’ vegetation referred to as jungles. We suggest, by analogy, that tropical regions in the Archean Eon were similarly overgrown with dense and ‘tangled’ microbial communities, such as the Balambano mat, that thus formed Precambrian continental jungles.

Combining cell counts and C-fixation rates allows us to evaluate the possible importance of subaerial microbial mats in driving evolutionary processes during the Archean Eon. At 10% surface coverage, microbial mats likely harboured tenfold fewer microbial cells, globally, than the Archean surface ocean (Supplementary Table 6 (ref. ^55^)). Based on our measurements in Balambano mat, global rates of cell division, on the other hand, could have been similar in the two ecosystems (Supplementary Fig. 7 and Supplementary Table 6), and this implies similar annual probability for genetic mutations on land and in the ocean. In microbial mats, cells also live in much closer proximity than in the open ocean, which fosters horizontal gene transfer, and thus rates of genetic exchange are expected to be higher in mat communities than in the ocean. Together, similar mutation rates and higher frequencies of horizontal gene transfer in land-based ecosystems would thus have made them prime locations for microbial evolution in the Archean Eon, implicating continental mats as possible drivers of the Archean expansion in biological diversity^46^.

Continental microbial communities have also been implicated in oxidative weathering, global oxygen budgets, and the evolution of atmospheric O_2_ and CH_4_ in the Precambrian Eons^44,56^. To directly test the potential role of continental mat communities in global O_2_ cycling, we partitioned net mat O_2_ sinks between loss to the atmosphere and consumption through oxidative weathering of the underlying bedrock (Supplementary Figs. 6, 8, and 9, and Table 1). Under the modern oxygenated atmosphere, 6.3 mmol m^−2^ d^−1^ or 16% of gross O_2_ production escapes the mat surface to the atmosphere, 6% is consumed through weathering of the underlying bedrock, and the balance channelled through respiration (Supplementary Figs. 6, 8, and 9, and Table 1). Under low-O_2_ early-to-mid-Archean atmospheres, 24 mmol m^−2^ d^−1^ or up to 70% of the gross O_2_ production would escape from the mat surface (Supplementary Figs. 6, 8, and 9, and Table 1). With minimal continental freeboard, such an O_2_ flux from continental mats would be entirely consumed through reactions with atmospheric O_2_ sinks even at 100% land coverage^44^ (Fig. 6). Growth of landmass in the late Archean to early Palaeoproterozoic to about 10% of the Earth surface^50^, however, could have allowed O_2_ effluxes from continental mats to overcome these sinks and O_2_ to accumulate in the atmosphere, if residual organic carbon was ultimately buried in marine sediments, even with minimal contribution from marine or benthic freshwater photosynthesis. Rates of organic carbon reoxidation would decrease upon export from the mat into surrounding anoxic environments^57^ promoting transport to the oceans, burial in sediments, and net oxygenation of the atmosphere. At the same time, CH_4_ effluxes from terrestrial microbial mats to the atmosphere could have contributed to atmospheric haze formation in the Archean Eon^58^ and climate warming throughout the Archean and Proterozoic Eons^56^. The combined effects of mat O_2_, organic C, and CH_4_ efflux should be investigated further, but overall the growth of continental landmass would have redistributed biogeochemical cycles between the oceans and continents with strong potential to alter the atmospheric chemistry between the late Archean and early Proterozoic Eons.

Increased fluxes of nutrients and redox-sensitive trace metals to the oceans 100’s of millions of years before the Great Oxidation Event (GOE)^5,59–61^ are used to infer high atmospheric oxygen levels in the late Archean Eon. Indeed, there is abundant evidence for the emergence of oxygenic photosynthesis by at least the Mesoarchean Era and this comes from both geochemical proxies^59,61,62^ and molecular phylogenies^25,63^. Rapid recycling of oxygen produced in terrestrial microbial mats through respiration or coupled to rock weathering and sulphide mineral oxidation, however, would have allowed for delivery of such redox tracers to the oceans even under an anoxic atmosphere^44^. It is thought that aerobic respiration may have evolved before oxygenic photosynthesis^64^, but alternatively could have emerged as the immediate response^65^. A 0.02% coverage of the late Archean land mass^50^ by continental photosynthetic microbial mats would have been sufficient to produce the sulphur flux proposed for the Mesoarchean, and at a 1% coverage, a modern sulphur flux can be sustained^5^ (Fig. 6). The presence and activity of nitrogen fixing and nitrifying bacteria in the mat also implies that terrestrial mats could have supplied the coastal ocean with oxidized N-species under an anoxic atmosphere, as previously suggested for biological soil crusts^43^. Nitrification likely emerged in response to oxygenic photosynthesis^66^, even though widespread evidence for nitrification at global scales seems only to emerge following the GOE^67^. Based on our measured net production and C:N ratios, the mats could have supplied 1 Tmol yr^−1^ nitrate to the coastal ocean and such a flux may be recorded in late Archean sedimentary N-isotope signals^68,69^. Mat-enhanced fluxes of nitrate and sulphate could also have stimulated coastal marine primary production, fuelling sulphate reduction and supporting the development of late Archean sulphidic oceans^43,70^.

Our work on Balambano mat qualitatively and quantitatively shows how oxygenic photosynthesis supports taxonomically and metabolically diverse microbial jungles that can drive fluxes of matter and energy at global scales. Export of nutrients from terrestrial mats to the coastal ocean can be sustained by aerobic metabolisms and weathering reactions that operate within the mat and largely independent of atmospheric oxygen. Terrestrial mat CH_4_ effluxes can contribute to atmospheric chemistry and climate, possibly playing an outsized role compared to marine communities in the Proterozoic Eon^56^. In this way, terrestrial microbial jungles would have established the first links between biogeochemical cycles on land and in the ocean during the Archean Eon and these links could have continued throughout the Proterozoic Eon until plants colonized the continents and usurped the microbial continental biosphere in the mid-Ordovician some 470 Ma.

## Methods

### Sampling site

Samples were collected from an outcrop of ultramafic bedrock exposed during the construction of the Balambano hydroelectric dam (Supplementary Fig. 1). The dam was constructed in 1997 and the samples collected in 2006 and again in 2009, 9–12 years after bedrock exposure. The outcrop measures several tens of square-meters and is periodically wet by direct precipitation as well as overland flow (Fig. 1a). The outcrop is mostly covered by the microbial mat, but bedrock is exposed in places, and is also partly covered by grasses. The maximum observed thickness of the mat was 6 cm, but typical observations were ~3 cm (Fig. 1b).

### Mat description

The microbial mat forms distinct layers that vary on a millimeter–centimeter scale (Fig. 1b). The mat is mechanically robust allowing removal of intact square-decimeter sections. The dominant colour of the mat is ochre, which is characteristic of iron oxyhydroxides. A green layer is visible at about 2–5 mm depth, coinciding with a peak in oxygen concentrations (Fig. 5 and Supplementary Fig. 10). Light microscopy revealed opaque minerals and rock fragments trapped in a translucent matrix that also hosted microbial filaments (Fig. 1c). Fluorescence microscopy of individual filaments revealed the presence of heterocystous cyanobacteria (Fig. 1d), enabling the mat to fix nitrogen from the atmosphere.

### Oxygen measurements

In situ profiles of oxygen concentrations were determined using a gold amalgam voltammetric microelectrode according to Brendel and Luther^71^ (Supplementary Fig. 10). The profile was measured in 2006 directly on the outcrop hosting the mat. These measurements are thus distinct from the measurements used to construct the profiles shown in Fig. 5, which were conducted in a greenhouse with a Clark type microelectrode. We used 25-µm-diameter Clark-type oxygen sensors (Unisense, DK) and m-profiler software (MPI-Bremen, D) for diel oxygen concentration profile measurements. Subsamples of the mats were transferred to a greenhouse in 2009 to conduct measurements of photosynthesis and respiration. These samples were kept in flumes in a greenhouse and oxygen profiles measured under subaerial conditions and within 1 month of collection. Profiles were measured every ~3 h over a 24-h cycle (Fig. 5a). Profiles measured at 3 a.m. and 3 p.m. were used to calculate respiration and net oxygenic photosynthesis, respectively, using a 1D reaction-transport model^72^ (Fig. 5b, c). The gross photosynthesis rate was calculated as the sum of the net rate and the night-time respiration. As respiration rates can be lower at night, due to decreased oxygen concentrations, this value represents the lower limit of gross photosynthesis in the mat.

### Flux determinations from profiles

Oxygen fluxes to both the atmosphere and the underlying rock were calculated based on oxygen concentration gradients using Fick’s law of diffusion:

1 $$J = - \phi \times {{D}}\frac{{\delta c}}{{\delta x}},$$

where *J* is the diffusive flux (mol m^−2^ y^−1^), *ϕ* the porosity, *D* the tortuosity-corrected diffusion coefficient (m^2^ y^−1^), and *δc*/*δx* the concentration gradient (mols m^−4^). The average porosity of the mat of 0.97 was used for all calculations. The flux to the rock was determined from an in situ profile measured with a voltammetric electrode (Supplementary Fig. 10), and the atmospheric flux from a profile measured by a Clark-type electrode in the greenhouse at a higher resolution (Fig. 5).

Oxygen fluxes from the mat to the atmosphere would be different under an anoxic atmosphere, like in the Archean Eon, than in the modern-day atmosphere with 20% oxygen. To estimate fluxes from a mat like Balambano to an anoxic atmosphere, we modelled oxygen production rates in the mat based on oxygen profiles (Supplementary Figs. 8 and 9). Oxygen concentrations were modelled as a function of rates of photosynthesis that are themselves influenced by light availability and rates of respiration, which in turn were affected by oxygen availability and described by Michaelis–Menten-type equations.

Oxygen production (*O*_2,Prod_) is modelled according to:

2 $${O}_{2,\,{\rm{Prod}}} = \frac{{{\rm{PAR}} \times V_{{\rm{max}},{\rm{Prod}}}}}{{\left( {K_{{\rm{m}},\,{\rm{Prod}}} + {\rm{PAR}}} \right)}}.$$

Photosynthetically active radiation (PAR) was set to 1500 µE m^−2^ s^−1^ at the surface decreasing by 1% every depth interval of 0.071 mm. *V*_max,Prod_ was set to 2.1 nM s^−1^; *K*_m,Prod_ is ‘the ‘affinity’ for light, set to 1000 µE m^−2^ s^−1^.

Respiration (*O*_2,Resp_) was modelled according to:

3 $$O_{2,\,{\rm{Resp}}} = \frac{{O_{2,\,n - 1,x} \times V_{{\rm{max}},\,{\rm{Resp}}}}}{{\left( {K_{{\rm{m}},\,{\rm{Resp}}} + O_{2,\,n - 1,x}} \right)}}.$$

*O*_2,*n*−1,*x*_ is the concentration at time *n*−1 (previous time step) and depth *x*, *V*_max,Resp_ was set to 1.75 nMs^−1^, and *K*_m,Resp_ was set to 250 nM of O_2_.

The diffussive flux (*F*_D_) from and to the adjacent depth (*x* ± 1) was modelled according to:

4 $$F_{{\rm{D}},\,x \pm 1} = \phi \times D\prime \times \frac{{O_{2,\,n - 1,\,x} - O_{2,\,n - 1,\, \pm 1}}}{{x - x_{ \pm 1}}},$$

where *ϕ* is the porosity (0.97), *D*′ the molecular diffusivity corrected for tortuosity of 1 × 10^−5^ cm^2^ s^−1^, *O*_2, *n*−1,*x*_ the O_2_ concentrations in the previous time step (*n*−1) at depth *x*, *O*_2, *t*=*n*−1,*x*±1_ the same at the depth above (*x*−1) or below (*x* + 1), *x* is the depth step that the concentration is modelled at, and *x*_±1_ is the depth step above (*x*−1) and below (*x* + 1).

O_2_ concentrations at time *n* and depth *x* are calculated in 1-s steps using the equation:

5 $$O_{2,\,n,\,x} = O_{2,\,n - 1,} + O_{2,\,{\rm{Prod}}} - O_{2,\,{\rm{Resp}}} - F_{{\rm{D}},\,x - 1} - F_{{\rm{D}},\,x + 1}.$$

The model was parameterized to reproduce the measured O_2_ profiles (Fig. 5b, c) and steady-state was approached within the first 1200 iterations of 1 s each (Supplementary Fig. 9). Next the oxygen concentration in the water was changed to 0 µM and the model run again with 1200 iterations of 1 s each (Supplementary Figs. 8 and 9) to reach a new steady-state.

The sensitivity of the model was tested by changing the variable parameters by ±10% (change of 0 and 5% for the decrease of PAR with depth) and evaluating the influence of these changes on the O_2_ slope across the mat–water interface at the end of the model run. This 10% change resulted in a change in the slope of ±3% for the high O_2_ scenario and less than ±1% for the no O_2_ scenario (Supplementary Table 7). Changes of the variable parameters had a much stronger effect on the oxygen concentrations deeper in the mat than they did on the slope at the mat–water interface.

Oxygen fluxes to the mat surface were calculated using Fick’s first law with the same constants noted above. Fluxes are 6.3 mmol m^−2^ d^−1^ measured in our mats under atmospheric oxygen concentrations. Under an anoxic atmosphere, the model fluxes increase to 24 mmol m^−2^ d^−1^, which is similar to the 13 mmol m^−2^ d^−1^ calculated for hypothetical Proterozoic mats by Zhao et al.^56^. Zhao et al. use a much more complex modelling approach discriminating between visible and near-infrared light intensities, variable substrate concentrations, inclusion of microbial growth and death rates, and internal methane cycling. The agreement between our observations and models and their models underscores the likely extensibility of our results.

### Methane flux determination

A subsample of the mat was incubated in the dark for 1.5 months and the methane production was measured in the headspace with a gas chromatograph (GC) equipped with an flame ionization detector (FID) (SRI, USA). An areal flux was determined assuming a 6-cm-thick mat. Half the flux determined by the incubation was used for the flux determinations to account for inhibition of methanogenesis under the oxic conditions that develop during the day. This flux is about 100-fold greater than the fluxes determined by Hoehler et al.^13^ in a modern, hypersaline microbial mat and 1/10 of fluxes modelled for hypothetical Proterozoic terrestrial mats^56^.

### Flux balance

The fluxes calculated through different approaches and considerations are compiled in a box model and converted into oxygen equivalents (normalized to 4-e^−^ transfers) for comparison (Supplementary Fig. 5). The oxygen flux to the rock and the atmosphere are modelled from in situ and greenhouse profiles, respectively. The greenhouse profiles were used to determine the atmospheric flux as they have a higher spatial resolution. OM production was calculated from the accumulation of a 6-cm-thick mat with 0.97 porosity and the profile of the wt% C (Supplementary Fig. 6), assuming accumulation over 9 years. The C-fixation rate was converted into O_2_ consumption based on a 4-e^−^ transfer from OM (CH_2_O) to CO_2_ and a 4-e^−^ transfer from O_2_ to 2H_2_O. The respiration rate was calculated from night profiles measured in the greenhouse, while the CH_4_ flux was determined from incubations of the mat in the dark.

### Organic C content and C, N isotopic compositions

The organic carbon (*C*_org_) content, and isotopic composition, as well as the N isotopic composition of the mat was measured through combustion-based elemental analysis of freeze-dried subsections on an elemental analyzer coupled to an isotope ratio mass spectrometer (EA-IRMS; Thermo delta plus). Inorganic carbon was removed by first washing (2×) for 24 h with a dilute HCl (1 M) solution. The absolute instrumental reproducibility of these analyses was determined from replicate measurements of Organic Analytical Standard substances (acetanilide, atropine, cyclohexanone-2,4-dinitrophenyl-hydrazone, and urea), and for concentrations estimated at ±0.1% for *C*_org_ and ±0.3% for *N*_TOT_ with a relative analytical reproducibility of 5%. Isotopic data are reported in the standard *δ* notation with respect to V-PDB for carbon^73^ and atmospheric N_2_ for nitrogen. The analytical uncertainty on isotope ratio determinations was ±0.1‰ for carbon and ±0.2‰ for nitrogen.

### Inorganic solid-phase composition

The mineralogy and solid-phase chemistry of the microbial mat was determined on freeze-dried sections as described in ref. ^74^. In brief, the mineralogy was analyzed using X-ray diffraction (XRD; Rigaku MiniFlex (Rigaku, The Woodlands, TX, USA) with Cu Kα radiation) (Supplementary Fig. 7) and the solid-phase chemistry using X-ray fluorescence (XRF) on a lithium tetraborate fused bead using a Philips PW2440 4 kW instrument (Panalytical Inc., Natick, MA, USA) (Supplementary Table 3). Accuracy for major and trace elements was within 1% and 5%, respectively, based on the analyses of standard reference materials, and relative precision was within 0.5% determined by repeated analyses of the same fused bead.

### Molecular microbiology

Samples for molecular microbiological analyses were subsampled in the field and frozen at −20 °C, shipped to Denmark on dry ice, and stored frozen until extraction. The microbial mat was aseptically sectioned into three layers: top (0–2 mm), middle (2–10 mm), and bottom (10–25 mm) and weighed. Microbial community DNA was extracted from ~0.25 mg of microbial mat using the Power soil® DNA Isolation Kit (MoBio Laboratories, Inc., Solana Beach, CA) according to the manufacturer’s protocol. The purity and quantity of DNA extracted were assessed and determined using both a NanoDrop 1000 spectrophotometer (Thermo Scientific) and the Picogreen® florescence assay.

### 16S rRNA gene amplicon sequencing and bioinformatic analysis

16S rRNA gene amplicon sequences were generated by the Joint Genome Institute (Walnut Creek, CA) through a community sequencing program project (CSP-2067). Libraries for Illumina MiSeq sequencing (a 2 × 300 bp reads configuration) were produced by amplifying region V4-V5 of the 16S rRNA gene using primers 515F and 926R. DNA from all samples was amplified by PCR in triplicate using barcoded primer pairs flanking the V4-V5 region of the 16S rRNA gene as previously described^75,76^. Sequences were processed using Mothur^77^ (https://www.mothur.org/wiki/MiSeq_SOP). Briefly, sequences were removed from the analysis if they contained ambiguous characters, had homopolymers longer than 8 bp, or did not align to a reference alignment of the correct sequencing region. Unique sequences were identified and their frequency in each sample determined and then a pre-clustering algorithm^78^ was used to further de-noise sequences within each sample^79^. Unique sequences were aligned against a SILVA reference alignment (available at http://www.mothur.org/wiki/Silva_reference_alignment). Sequences were chimera checked using UCHIME^80^ and reads were then clustered into OTUs with 97% sequence identify using OptiClust^81^. OTUs were classified using SILVA reference taxonomy database (release 132, available at http://www.mothur.org/wiki/Silva_reference_files) (Supplementary Fig. 8, Supplementary Table 4). Diversity metrics were calculated in Mothur.

### qPCR and DNA content

Total bacterial 16S rRNA gene copy numbers were determined by qPCR using a bacterial-specific (27F, 5′-AGAGTTTGATCCTGGCTCAG) forward primer coupled to a universal reverse primer (DW519R, 5′-GNTTTACCGCGGCKGCTG) according to Zaikova et al.^82^ (Supplementary Table 5). Standards for total bacteria quantification were derived from 16S rRNA gene clone libraries using a 16S rRNA from the bacteria SUP05 according to Zaikova et al.^82^. A tenfold dilution series for each standard ranging from 3 × 10^2^ to 3 × 10^7^ copies μl^−1^ for bacteria was used in real-time analysis. For the purposes of this study, the limit of detection was set at the Ct values of the no template controls and was determined to be ∼5 × 10^1^  copies  μl^−1^ for all sample replicates. The quantity of DNA extracted was also used to estimate cell abundance assuming 4 fg of DNA per cell.

### Cell counts

Cell abundances were determined using epifluorescence microscopy. The mat was aseptically sectioned into three layers: top, middle, and bottom, weighed and then preserved with 2.5% (final concentration) of glutaraldehyde. The fixed samples were washed twice with phosphate-buffered saline and then dissociated form the mat matrix according to Morono et al.^83^. In brief, mat sections were immersed in a solution of 2.5% NaCl, detergent mix (100 mM EDTA, 100 mM sodium pyrophosphate, 1% (v/v) Tween 80), and methanol, then vigorously shaken for 120 min at 500 rpm. After shaking, the mat sections were sonicated and then carefully layered onto a 50% (w/v) Nycodenz solution^84^. Samples were centrifuged at 4500 × *g* for 30 min after which the supernatant, including the high-density layer(s), was carefully removed and transferred to a separate vial. Cells were visualized following staining with DAPI using an eipfluorescence microscope (Zeiss, axioscope) with a 40× objective lens and enumerated using a bright line counting chamber (Hausser scientific) (Supplementary Table 5). The mat consisted of a large number of small bacterial cells and a relatively small number of larger, filamentous microbes. The DAPI counts only represent the small bacterial cells, as the larger filaments were not separated from the matrix. The general agreement between DAPI counts and q-PCR, as well as quantification of DNA yields, gives us confidence that visual enumeration captured the vast majority of the cells. Our cell counts lie at the lower end of previously reported values for mats^85^, and this is in line with the minimum values we report.

### Calculation of cellular carbon and cell divisions in subaerial mats

To calculate cellular biomass, we considered cell abundances of 10^8^–10^9^ cells cm^−3^ and cellular carbon contents of 1–150 fg C cell^−1^ (refs. ^86–88^). This translates to between 10^−7^ and 1.5 × 10^−4^ g C cm^−3^ of wet mat. Based on a porosity of 0.97 and a particle bulk density of 2.65 g cm^−3^, the total organic carbon content of 1–12% dry mass translates to carbon concentrations in the wet mat of between 0.7 × 10^−4^ and 9.5 × 10^−3^ g C cm^−3^. This means that cellular biomass accounts for between 0.001 and 20% of the total organic carbon. Cell counts (Supplementary Table 6) and gross photosynthesis rates (Fig. 5) were used to calculate a cell turnover time in the mats based on 1–150 fg of C per cell^86–88^. Cell counts of the top section were used, as the locus of photosynthesis is within this depth interval (Fig. 5b). This resulted in a turnover time of 0.2 d or 5 divisions per cell per day. Based on the range in C concentrations per cell, this results in 6.9 × 10^22^ to 1.0 × 10^25^ divisions per day for 0.1% continental coverage by microbial mats 6.9 × 10^25^ to 1.0 × 10^28^ divisions per day for 100% continental coverage (Supplementary Fig. 10, Supplementary Table 7). Archean ocean surface area was calculated based on a land area of 5% leaving an ocean area of 95% of the earth surface or 485 × 10^6^ km^2^. Using cell densities and turnover times for the surface ocean according to Whitman et al.^55^ and a tenfold lower primary productivity than the modern results in 4.8 × 10^27^ cells and 2–8 × 10^26^ divisions per day (Supplementary Fig. 10, Supplementary Table 7).

### Eukaryotes

Microeukaryotes, including diatoms and algae, were visible through microscopy, but were not directly analyzed through, for example, amplicon sequencing of the 18S rRNA gene or ITS regions. 16S rRNA gene sequences recovered from mitochondria and chloroplasts, however, were rare in our amplicon sequence data and this is at least qualitatively consistent with our limited observations of Eukaryotes through microscopy. For example, chloroplast reads represented <0.2% of all reads and <2% of all photosynthetic reads (Supplementary Table 8). Photosynthesis and respiration by microeukaryotes would have been integrated into our rate measurements, but their likely very low abundance, compared to bacteria, implies that they play a limited role. We do recognize, however, that abundance is not a good proxy for activity, but given the extreme dominance of bacteria here, we argue that they also play the most important role.

## Supplementary information

Final SI PDF

## Data Availability

Sequence data that support the findings of this study have been deposited into the SRA database under SRA accession: SRP155785. Additional data that support the findings of this study are available from the corresponding author upon request.
