# Supplementary material for: Mesophilic microorganisms build terrestrial mats analogous to Precambrian microbial jungles
Source: Nat Commun. 2019 Sep 20;10:4323. doi: 10.1038/s41467-019-11541-x (PMC6754388; doi:10.1038/s41467-019-11541-x)
Supplement: Supplementary file 1 — Final SI PDF [file 41467_2019_11541_MOESM1_ESM.pdf]

Supplementary information

Mesophilic microorganisms build terrestrial mats analogous to Precambrian microbial jungles

N Finke, RL Simister, AH O'Neil, S Nomosatryo, C Henny, LC MaClean, DE Canfield, K Konhauser, SV Lalonde, DA Fowle, and SA Crowe

Supplementary Figures

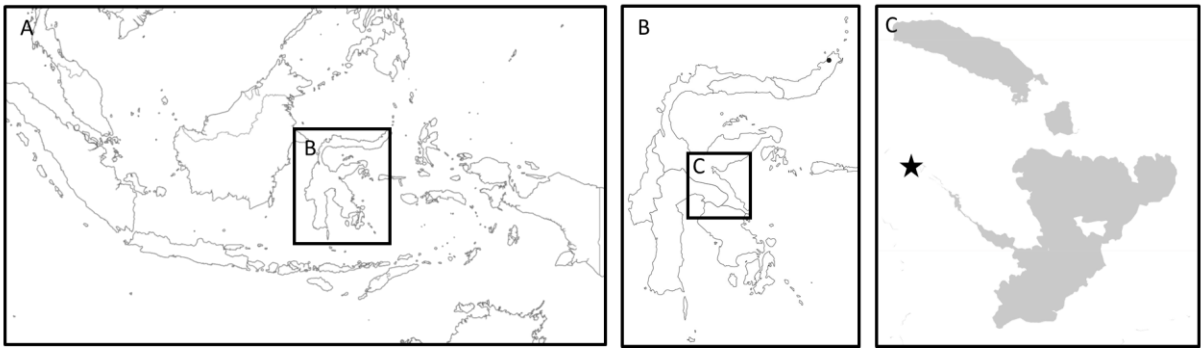

Supplementary Figure 1: (a) map of Indonesia, (b) map of Sulawesi Island, and (c) map of the Malili lake system, with the Balambano hydroelectric dam indicated with a star. The dam is to the west of Lake Towuti on the Larona river.

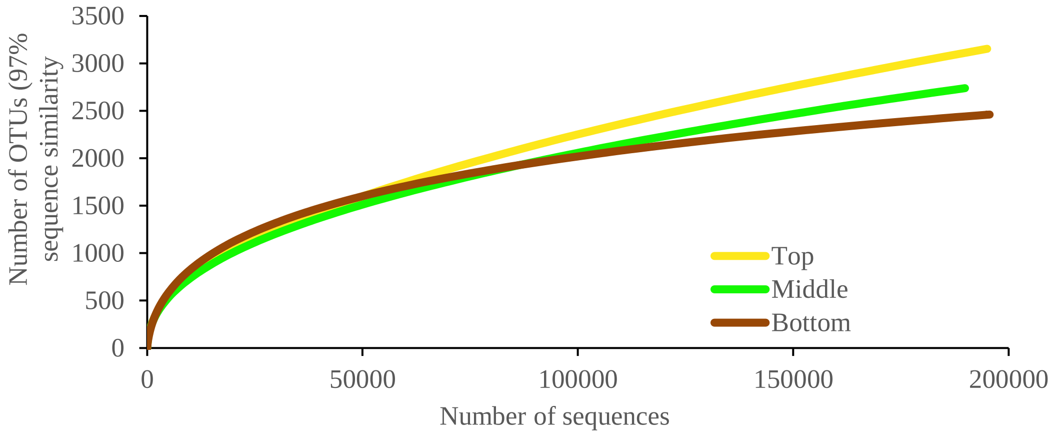

Supplementary Figure 2: Microbial diversity of microbial mat sections. Rarefaction curves are based on OTUs at 97% sequence similarity. Calculations were performed in Mothur (Schloss et al., 2009).

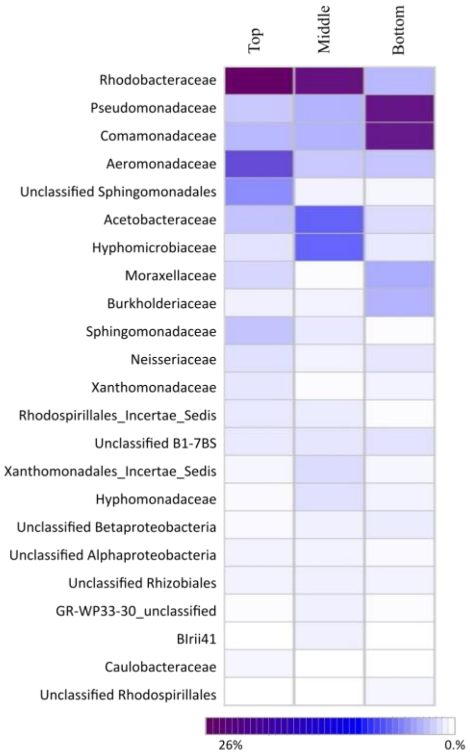

Supplementary Figure 3: Relative abundance of the top 25 most abundant proteobacterial taxa classified at the family level in 3 sections of the microbial mat (Top = 0-2mm; Middle = 2-5mm; Bottom = 5-20mm). Dark purple squares represent the most abundant family's and white squares represent the lowest abundant families.

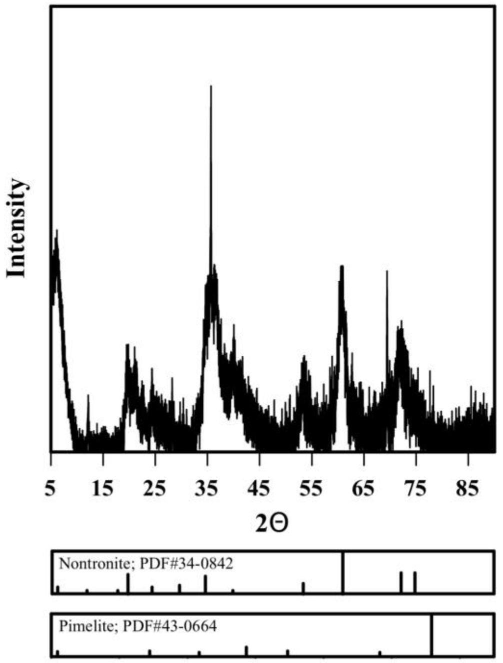

Supplementary Figure 4 XRD analysis of the microbial mat in comparison to the clay minerals nontronite and pimelite.

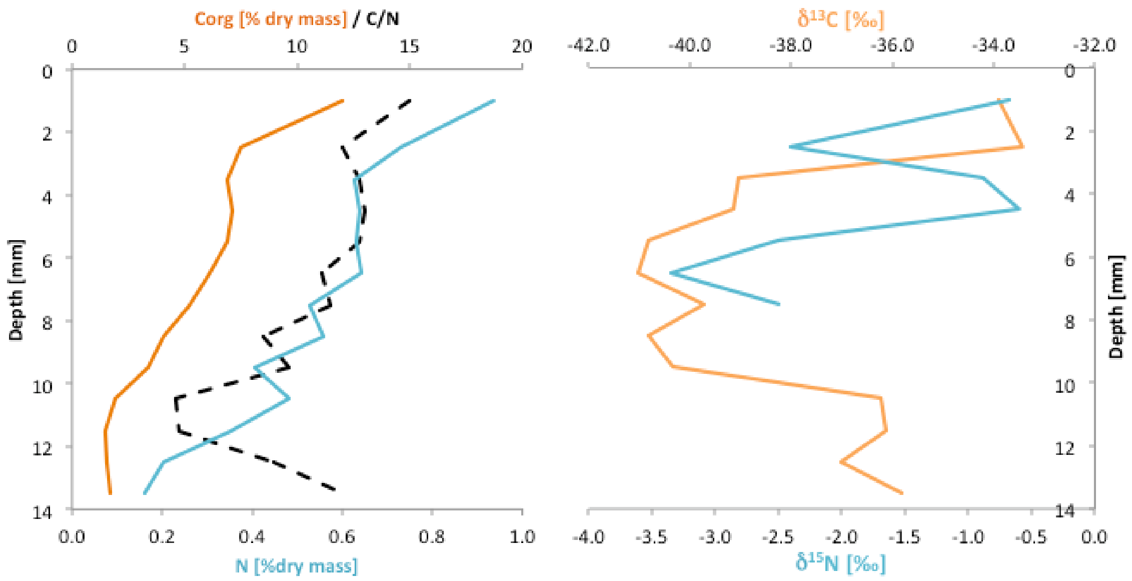

Supplementary Figure 5: Elemental and isotopic composition of the mat.

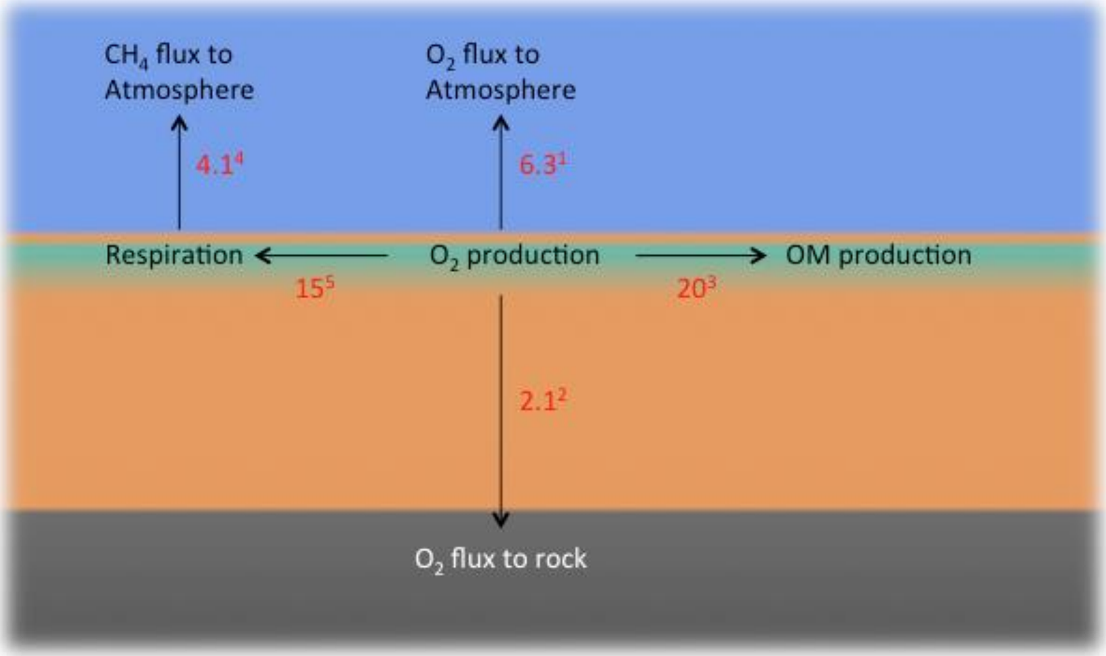

Supplementary Figure 6: Box model of fluxes of oxygen equivalents in the microbial mat. O_2 produced in the mat is either consumed by respiration or weathering of the underlying reduced rock, or escapes to the atmosphere. The organic matter produced by oxygenic photosynthesis is either accumulating in the mat or respired in part involving methane production. O_2 production resulting from these fluxes are presented in Table 1. Superscript numbers refer to the method used for flux determination with fluxes reported in mmol m^{-2} d^{-1} ([1] flux determination from daytime greenhouse profile = 6.3, [2] 1D transport reaction modeling of in situ profile = 2.1, [3] in situ mat accretion = 20, [4] headspace analysis in dark mat incubation = 4.1, [5] 1D transport reaction modeling of night time greenhouse profiles = 15).

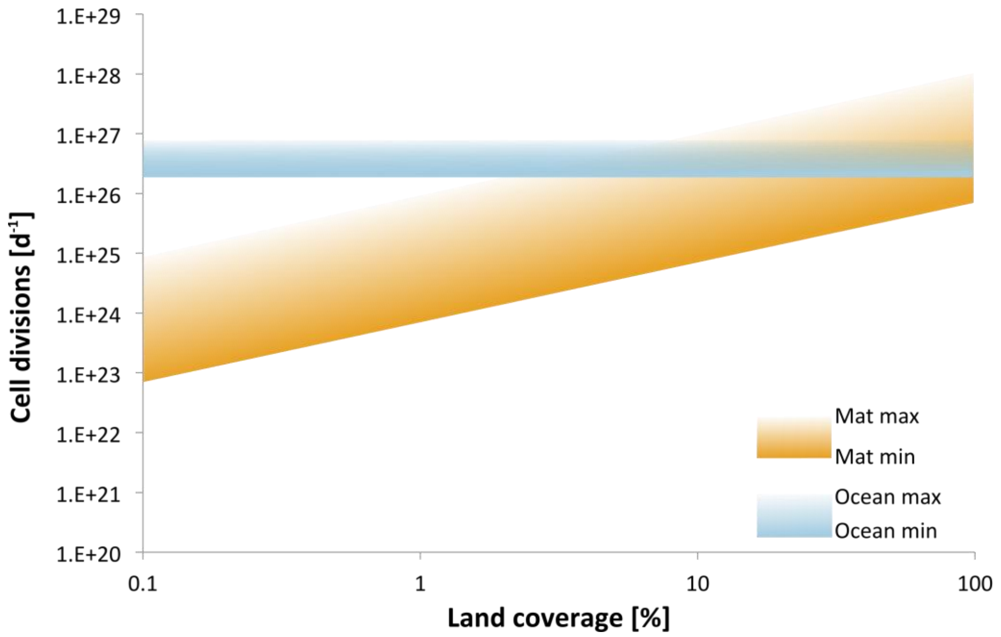

Supplementary Figure 7: Cell divisions calculated on a global scale based on cell counts in the mat and estimates for surface ocean according to [28] scaled by Archean land mass covered by subaerial mats.

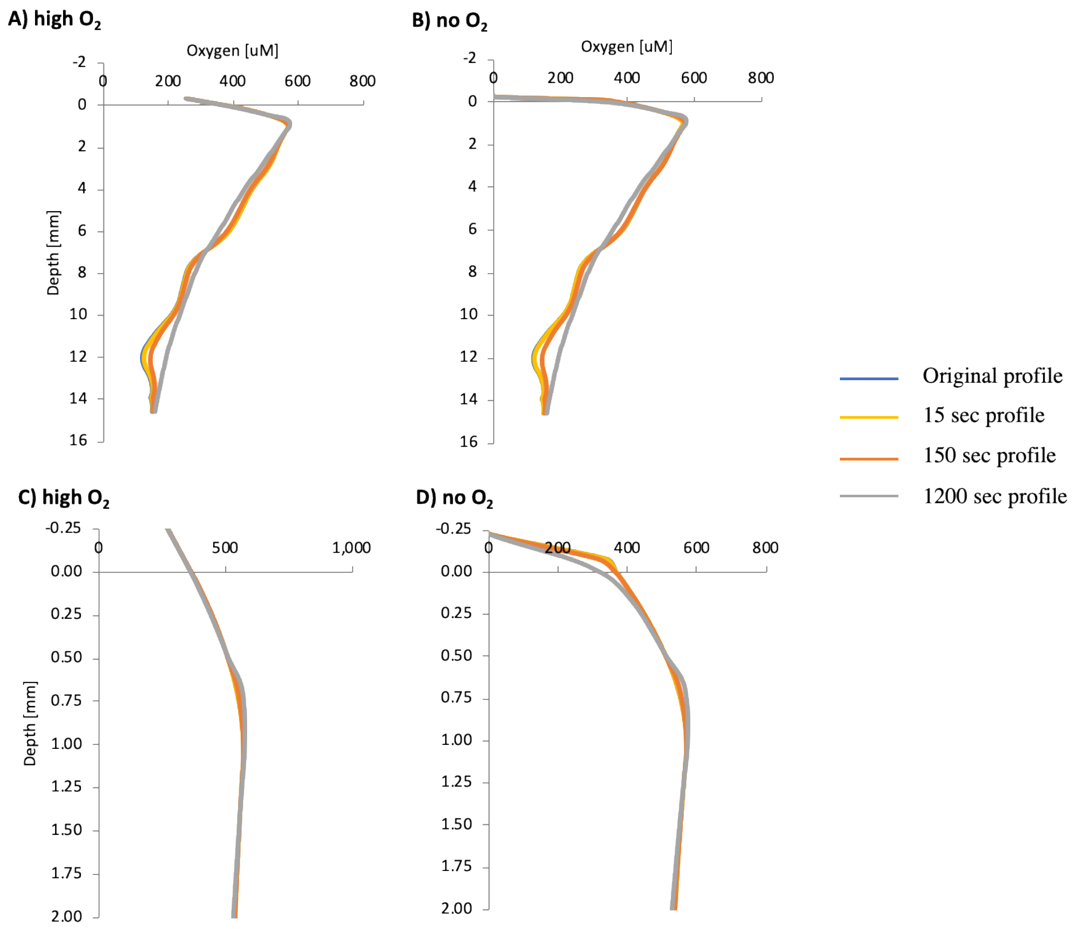

Supplementary Figure 8: Oxygen profiles from 1D modeling of greenhouse-based measurements. "Original profile" represents the oxygen profile measured in the mat. The model was run for up to 1200 seconds and modeled profiles from 3 different time points are shown. Results are shown for measured overlying oxygen concentrations (A, C) and for the overlying oxygen set to 0 µM (B, D). Panels C and D show a close up of the top 2mm of the mat and the mat water interface.

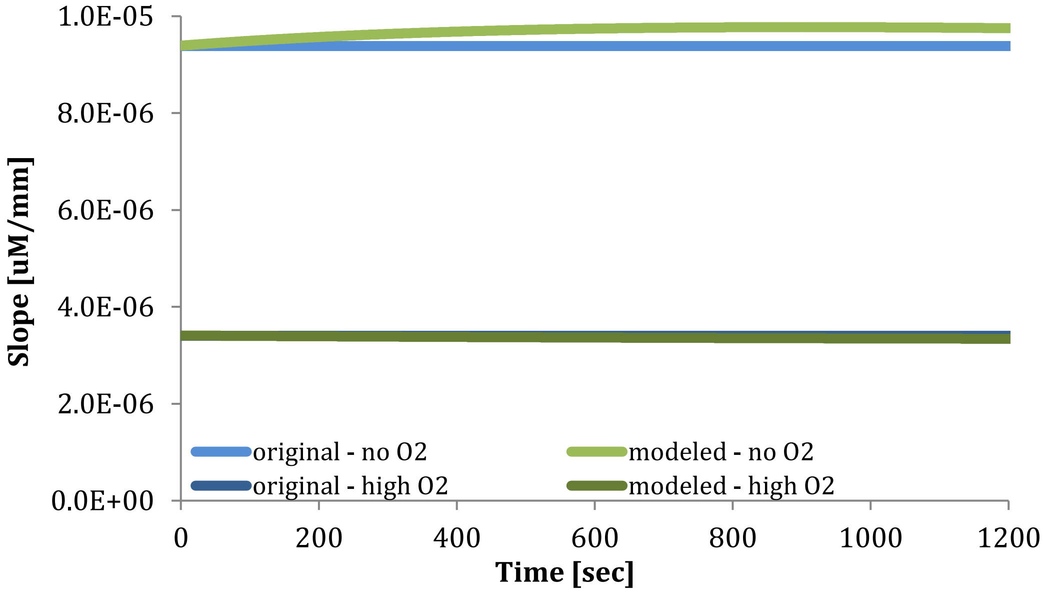

Supplementary Figure 9: Changes in oxygen slope across the mat-water interface over the course of the 1200 seconds of the modeling. Overlying water oxygen concentrations were fixed at either measured values (high O_2) or set to 0 µM (no O_2). "original – high O_2" represents the slope measured in the mat, "original – no O_2" the slope when changing the original profile to 0 µM oxygen in the water column. There is a slight increase in the slope in the "modeled – no O_2" slope at first, but the slope has stabilized at 1200 seconds, which is why this time is presented in Supplementary Figure 8.

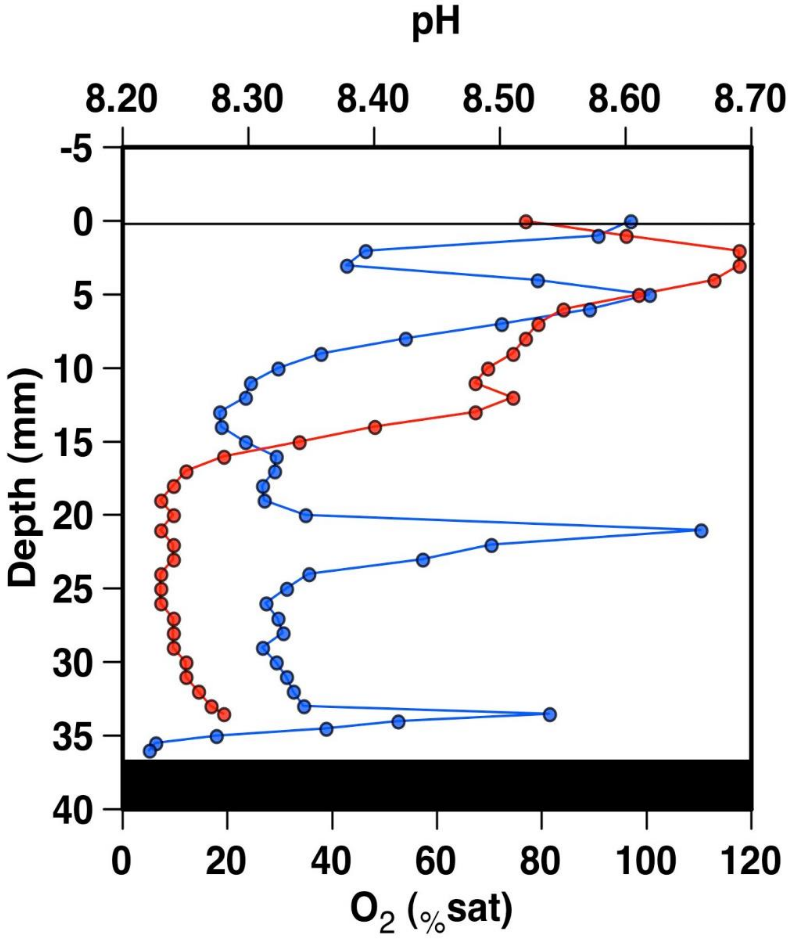

Supplementary Figure 10: In situ oxygen (blue) and pH (red) profiles from the Balambano mat. The black area at the bottom represents the ultramafic rock the mat is growing on.

Supplementary Tables

Supplementary Table 1: Cell abundance in the mat determined by 3 different methods.

	Cell count	qPCR 1	qPCR 2	ng DNA	Average	Standard Error
Top	2.52E+08	1.94E+08	9.87E+08	3.33E+09	1.19E+09	7.35E+08
Middle	1.86E+08	3.87E+07	2.80E+08	7.19E+08	3.06E+08	1.46E+08
Bottom	1.91E+08	2.75E+06	1.87E+07	1.58E+08	9.27E+07	4.79E+07

Supplementary Table 2: Summary of sequence and diversity statistics.

Sample	# seqs	rarefied #seqs	Coverage	Observed OTUs	Chao1
Top	195009	189856	0.99	3111	6343
Mid	189856	189856	0.99	2739	4855
Bottom	195580	189856	1.0	2441	3254

Supplementary Table 3: Total number of 16S-rRNA reads for microbial taxa with important inferred ecological roles and/or relatively high abundance across the three mat sections and their phylogenetic affiliation.

Number of Sequences			Phylogenetic affiliation
top	middle	bottom	
0	3	9	Euryarchaeota; Methanomethylovorans
16	3	332	Thaumarchaeota; Candidatus Nitrososphaera
7	11	9	Bathyarchaeota (formally MCG group)
7	2	9	Alphaproteobacteria; Methylobacteriaceae
1	4	0	Alphaproteobacteria; Rhizobium
25	19	21	Alphaproteobacteria; Azospirillum
14	197	9	Gammaproteobacteria; Azomonas
0	1	6	Betaproteobacteria; Nitrosomonadaceae
50	520	31	Verrucomicrobia:Candidatus Methylacidiphilum
58	3	0	Cyanobacteria; Haloleptolyngbya
9062	20334	865	Cyanobacteria; Leptolyngbya
54	0	0	Cyanobacteria; Pseudoanabaena
8339	5024	642	FamilyI_unclassified
838	2171	413	Chloroflexi; Roseiflexus
1311	1724	61	Chloroflexi; Candidatus Chloroploca
597	1230	21	Chloroflexi; Chloronema
42	72	1592	Nitrospirae; Nitrospira
1775	552	3477	Firmicutes: paenibacillaceae
2377	2173	39059	Firmicutes: unclassified bacillales
950	848	150	Firmicutes: Clostridiaceae
2826	1836	332	Firmicutes: Veillonellaceae

Supplementary Table 4: Solid phase composition of the Balambano microbial mat in comparison to Harzburgite and saprolite measured by XRF.

	Balambano site	
**Element**	**Harzburgite**	**Microbial Mat**
SiO_2	43.52	39.24
TiO_2	0.07	0.04
Al_2O_3	2.53	1.28
Fe_2O_3	9.00	9.39
MnO	0.12	0.161
MgO	37.41	19.08
CaO	2.16	0.21
Na_2O	0.07	0.24
K_2O	0.01	0.04
P_2O5	0.01	0.02
Co	0.0100	0.0131
Cr_2O_3	0.4068	0.2439
Ni	0.2171	0.3822
Sc	0.0015	0.0007
V	0.0061	0.0026
Zn	0.0012	0.0072

Supplementary Table 5: Literature values of maximum and average volumetric as well as areal oxygenic photosynthesis rates in microbial mats from different environments around the world. Gross rates reported if not noted otherwise

**max rate** **nmol cm^{-3} s^{-1}**	**average rate** **nmol cm^{-3} s^{-1}**	**Areal mmol m^{-2} d^{-1}**	**mat type**	**Origin**	**Reference**
0.86	0.10	23[a]/38	Subareal, mesophilic mat	Sulawesi Island, Indonesia	This study
18	8	1200	Hypersaline saltern, 1990	Guerrero Negro, Mexico	[1]
5	3	290	Hypersaline saltern, 1989	Guerrero Negro, Mexico	[1]
10	5		Hypersaline saltern, Pond 5	Guerrero Negro, Mexico	[2]
1	1		Hypersaline saltern, Pond 6	Guerrero Negro, Mexico	[2]
		140	Hypersaline saltern	Guerrero Negro, Mexico	[3]
11	6		Thermophilic mat	Yellowstone, USA	[4]
0.50			Pure culture from sulfidic spring	Frasassi Cave System, Italy	[5]
		6	Limnic mat	Antarctic Lake Fryxell	[6]
0.017	0.006		Limnic mat	Lake Sinai, Egypt	[7]
6	2	370	Lagoon	New Caledonia, France	[8]
8	4		Hypersaline saltern	Guerrero Negro, Mexico	[9]
9	5		Hypersaline salt marsch	Egypt	[10]
4	1		Hypersaline saltern	Chriprana, Spain	[11]
0.14	0.07	72	Gypsum crust, hypersaline saltern, 200 psu	Eilat Israel	[12]
0.02	0.01	14	Gypsum crust, hypersaline saltern, 230 psu	Eilat Israel	[12]
		14	Net rate, Iron rich mat, Camargue, France	Camargue, France	[13]
1.3[a, b]		140[a, b]	Benthic, limnic mat	Antarctica	[14]
0.38[a, b]		24[a, b]			
		68 [a, b]	Subtidal biofilm	Tanzania	[15]
		24[b]	Hypersaline saltern	Guerrero Negro, Mexico	[16]
0.023	0.005		Hypersaline saltern	Eilat, Israel	[17]
0.03	0.01		Hypersaline mat	Mallorca, Spain	[17]
6			Intertidal mat	Abu Dhabi, United Arab Emirates	[18]
13			Hypersaline lake in sabkha	Qatar	[19]
0.038 [a]			Benthic, limnic mat, 8.1m	Antarctica	[20]
0.02 [a]			Benthic, limnic mat, 16.4m	Antarctica	[20]
11	5	220	Hypersaline lake	Spain	[21]
0.06			Benthic, limnic mat	Antactica	[22]
3 [a, b]			Hypersaline saltern	Guerrero Negro, Mexico	[23]
13	5		Artificial lab mat		[24]
6.5 [a]	3.0 [a]		Stream shore	Antarctica	[25]
6			Hypersaline pond	Montpelier, France	[26]
15	7	860 [a]	intertidal flat in sabkha, 65 psu	Abu Dhabi, United Arab Emirates	[27]
2	1	170 [a]	intertidal flat in sabkha, 200 psu	Abu Dhabi, United Arab Emirates	[27]

[a] net rates reported [b] primary production reported as C fixation

Supplementary Table 6: Cell numbers and divisions in Archean subaerial microbial mats and surface oceans. Min and Max numbers for the mat division are based on 1 and 150 fg C per cell, respectively, and on 6 and 25 d turnover times for the ocean divisions.

Land coverage [%]	Land area [m^2]	Mat cells	Mat divisions [d^{-1}]		Surface ocean cells	Surface ocean divisions [d^{-1}]	
			min	max		min	max
100	2.6E+13	1.8E+27	6.9E+25	1.0E+28	4.8E+27	1.9E+26	8.0E+26
10	2.6E+12	1.8E+26	6.9E+24	1.0E+27	4.8E+27	1.9E+26	8.0E+26
1	2.6E+11	1.8E+25	6.9E+23	1.0E+26	4.8E+27	1.9E+26	8.0E+26
0.1	2.6E+10	1.8E+24	6.9E+22	1.0E+25	4.8E+27	1.9E+26	8.0E+26

Supplementary Table 7: Effect of 10% change in variables (for PAR decrease change from 1% attenuation to 0 or 5% are presented) on the O_2 flux across the mat water interface for the original profile (High O_2) and the anoxic atmosphere (no O_2). Values are reported as ratio between the original slope and the slope after changing the variable. PP stands for photosynthetic oxygen production.

	High O_2		No O_2	
	10% / 0% PAR decrease	-10% / 5% PAR decrease	10% / 0% PAR decrease	-10% / 5% PAR decrease
PAR	1.01	0.994	1.00	0.999
Km PP	0.995	1.01	1.00	1.00
Km resp	1.01	0.997	1.00	1.00
Vmax PP	1.03	0.982	1.00	0.998
Vmax resp	0.983	1.02	0.998	1.00
PAR decrease	1.01	0.989	1.00	0.999

Supplementary Table 8: counts and fraction of total, cyanobacterial and algae chloroplast 16S reads in our libraries.

	Total	Cyanobacterial	algal
	580445	54703	783
% algae	0.1	1.4	

Supplementary References

1. Canfield DE, Marais DJD. Biogeochemical Cycles of Carbon, Sulfur, and Free Oxygen in a Microbial Mat. *Geochim Cosmochim Acta* **57**, 3971-3984 (1993).

2. Jørgensen BB, Desmarais DJ. Competition for Sulfide among Colorless and Purple Sulfur Bacteria in Cyanobacterial Mats. *FEMS Microbiol Ecol* **38**, 179-186 (1986).

3. Bebout BM*, et al.* Methane production by microbial mats under low sulphate concentrations. *Geobiology* **2**, 87-96 (2004).

4. Ward DM, Ferris MJ, Nold SC, Bateson MM. A natural view of microbial biodiversity within hot spring cyanobacterial mat communities. *Microbiol Mol Biol Rev* **62**, 1353-+ (1998).

5. Klatt JM, Haas S, Yilmaz P, de Beer D, Polerecky L. Hydrogen sulfide can inhibit and enhance oxygenic photosynthesis in a cyanobacterium from sulfidic springs. *Environ Microbiol* **17**, 3301-3313 (2015).

6. Sumner DY, Hawes I, Mackey TJ, Jungblut AD, Doran PT. Antarctic microbial mats: A modern analog for Archean lacustrine oxygen oases. *Geology* **43**, 887-890 (2015).

7. Revsbech NP, Jørgensen BB. Photosynthesis of Benthic Microflora Measured with High Spatial-Resolution by the Oxygen Microprofile Method - Capabilities and Limitations of the Method. *Limnol Oceanogr* **28**, 749-756 (1983).

8. Pringault O, de Wit R, Camoin G. Irradiance regulation of photosynthesis and respiration in modern marine microbialites built by benthic cyanobacteria in a tropical lagoon (New caledonia). *Microb Ecol* **49**, 604-616 (2005).

9. Finke N, Hoehler TM, Polerecky L, Buehring B, Thamdrup B. Competition for inorganic carbon between oxygenic and anoxygenic phototrophs in a hypersaline microbial mat, Guerrero Negro, Mexico. *Environmental Microbiology* **15**, 1432-1550 (2013).

10. Glud RN, Kuhl M, Kohls O, Ramsing NB. Heterogeneity of oxygen production and consumption in a photosynthetic microbial mat as studied by planar optodes. *J Phycol* **35**, 270-279 (1999).

11. Jonkers HM*, et al.* Structural and functional analysis of a microbial mat ecosystem from a unique permanent hypersaline inland lake: 'La Salada de Chiprana' (NE Spain). *FEMS Microbiol Ecol* **44**, 175-189 (2003).

12. Canfield DE, Sørensen KB, Oren A. Biogeochemistry of a gypsum-encrusted microbial ecosystem. *Geobiology* **2**, 133-150 (2004).

13. Wieland A, Zopfi J, Benthien A, Kuhl M. Biogeochemistry of an iron-rich hypersaline microbial mat (Camargue, France). *Microb Ecol* **49**, 34-49 (2005).

14. Vincent WF, Castenholz RW, Downes MT, Howardwilliams C. ANTARCTIC CYANOBACTERIA - LIGHT, NUTRIENTS, AND PHOTOSYNTHESIS IN THE MICROBIAL MAT ENVIRONMENT. *J Phycol* **29**, 745-755 (1993).

15. Lugomela C, Soderback E, Bjork M. Photosynthesis rates in cyanobacteria-dominated sub-tidal biofilms near Zanzibar, Tanzania. *Estuar Coast Shelf Sci* **63**, 439-446 (2005).

16. Rothschild LJ. A MODEL FOR DIURNAL PATTERNS OF CARBON FIXATION IN A PRECAMBRIAN MICROBIAL MAT BASED ON A MODERN ANALOG. *Biosystems* **25**, 13-23 (1991).

17. Grotzschel S, de Beer D. Effect of oxygen concentration on photosynthesis and respiration in two hypersaline microbial mats. *Microbial Ecology* **44**, 208-216 (2002).

18. Al-Najjar MAA, de Beer D, Jorgensen BB, Kuhl M, Polerecky L. Conversion and conservation of light energy in a photosynthetic microbial mat ecosystem. *Isme J* **4**, 440-449 (2010).

19. Al-Thani R*, et al.* Community Structure and Activity of a Highly Dynamic and Nutrient-Limited Hypersaline Microbial Mat in Um Alhool Sabkha, Qatar. *Plos One* **9**, (2014).

20. Vopel K, Hawes I. Photosynthetic performance of benthic microbial mats in Lake Hoare, Antarctica. *Limnol Oceanogr* **51**, 1801-1812 (2006).

21. Ludwig R, Pringault O, de Wit R, de Beer D, Jonkers HM. Limitation of oxygenic photosynthesis and oxygen consumption by phosphate and organic nitrogen in a hypersaline microbial mat: a microsensor study. *FEMS Microbiol Ecol* **57**, 9-17 (2006).

22. Hawes I, Giles H, Doran PT. Estimating photosynthetic activity in microbial mats in an ice-covered Antarctic lake using automated oxygen microelectrode profiling and variable chlorophyll fluorescence. *Limnol Oceanogr* **59**, 674-688 (2014).

23. Houghton J, Fike D, Druschel G, Orphan V, Hoehler TM, Des Marais DJ. Spatial variability in photosynthetic and heterotrophic activity drives localized delta

C-13(org) fluctuations and carbonate precipitation in hypersaline microbial mats. *Geobiology* **12**, 557-574 (2014).

24. Bernstein HC*, et al.* Trade-offs between microbiome diversity and productivity in a stratified microbial mat. *Isme J* **11**, 405-414 (2017).

25. Fernandez-Valiente E, Camacho A, Rochera C, Rico E, Vincent WF, Quesada A. Community structure and physiological characterization of microbial mats in Byers Peninsula, Livingston Island (South Shetland Islands, Antarctica). *FEMS Microbiol Ecol* **59**, 377-385 (2007).

26. Lassen C, Glud RN, Ramsing NB, Revsbech NP. A method to improve the spatial resolution of photosynthetic rates obtained by oxygen microsensors. *J Phycol* **34**, 89-93 (1998).

27. Abed RMM, Kohls K, de Beer D. Effect of salinity changes on the bacterial diversity, photosynthesis and oxygen consumption of cyanobacterial mats from an intertidal flat of the Arabian Gulf. *Environ Microbiol* **9**, 1384-1392 (2007).

28. Whitman WB, Coleman DC, Wiebe WJ. Prokaryotes: The unseen majority. *Proceedings of the National Academy of Sciences of the United States of America* **95**, 6578-6583 (1998).
